# Risk prediction models for frailty in stroke patients: a systematic review and meta-analysis

**DOI:** 10.3389/fneur.2026.1804341

**Published:** 2026-04-09

**Authors:** Lingling Guo, Yi He, Wen Tang, Wanxi Zhu

**Affiliations:** 1 Department of Rehabilitation Medicine, the Second Affiliated Hospital of Chongqing Medical University, Chongqing, China; 2 Chongqing Geriatric Specialized Nursing Clinical Diagnosis and Treatment Center, Chongqing, China

**Keywords:** frailty, meta-analysis, risk prediction model, stroke, systematic review

## Abstract

**Objective:**

To systematically review the frailty risk prediction models for stroke patients.

**Data sources:**

Eight databases including PubMed, Web of Science, the Cochrane Library, Embase, CINAHL, CNKI, Wanfang Database, VIP and SinoMed were systematically searched from the inception of the databases to May 31, 2025.

**Study selection:**

Studies were screened independently by two researchers with systematic evidence-based training. In this review, We extracted data based on Data Extraction for Systematic Reviews of Prediction Modeling Studies (CHARMS).

**Data extraction:**

Two researchers independently screened the literature and extracted study data. We employed the Prediction Model Risk of Bias Assessment Tool (PROBAST) to assess both the risk of bias and applicability of the included studies.

**Data synthesis:**

A total of 19 studies and 24 frailty risk prediction models for stroke patients were included. The area under the Receiver operating Characteristic curve (AUC) of the included models ranged from 0.629 to 0.940, with 21 models having an AUC > 0.7. The combined AUC value of the 10 validation models was 0.87. However, all studies were at high risk of bias according to PROBAST, suggesting this estimate is likely inflated due to methodological weaknesses. The most frequently used predictors in the included models were age, activities of daily living, and NIHSS score.

**Conclusion:**

Current frailty prediction models for stroke patients have methodological weaknesses and high risk of bias. All included studies were from China (2023–2025), severely limiting generalizability to other populations. The pooled AUC (0.87) is likely inflated and should be interpreted cautiously. Rigorous external validation in diverse settings is needed before clinical use. Future studies should expand the sample size, strictly carry out study design and carry out multi-center external validation.

**Systematic review registration:**

https://www.crd.york.ac.uk/PROSPERO/view/CRD420251075547.

## 1 Introduction

Stroke is an acute cerebrovascular disease characterized by focal neurological function impairment caused by sudden rupture or blockage of blood vessels in the brain. It has five major features: high incidence, high disability rate, high recurrence rate, high mortality rate and high economic burden. It affects nearly 14 million people worldwide annually and results in approximately 120 million disability events (1–3). Research indicates that frailty is prevalent among stroke patients. At least one in four individuals who experience a stroke are found to be living with frailty (4). Frailty is an age-related geriatric syndrome characterized by a cumulative decline in physical function resulting from reduced physiological reserves and diminished resistance to stressors. It further increases the risk of adverse health outcomes (5–7). Research indicates that frailty is associated with adverse outcomes including falls, disability, and mortality (8). Frailty can be categorized into two stages: pre-frailty and frailty (9). The level of pre-frailty in stroke patients is associated with stroke severity and may elevate the risk of stroke recurrence (10). Moreover, frailty is independently linked to mortality within 28 days following ischemic stroke, with patients experiencing progressive deterioration in quality of life (11, 12). Evidence suggests that frailty may be reversed or delayed through various interventions, including physical activity, nutritional support, and cognitive training (13, 14). Therefore, as an independent predictor of post-stroke prognosis, assessing frailty risk in stroke patients is essential.

A risk prediction model is a statistical tool that estimates the likelihood of specific outcomes by integrating multiple variables, such as clinical indicators, biochemical markers, and imaging findings. The frailty risk prediction model for stroke patients enables healthcare professionals to identify individuals at higher risk of frailty and implement timely interventions based on different levels of risk stratification (15). Currently, the onset age of stroke has shown a trend toward younger populations, and frailty can occur across various adult age groups (16). Therefore, early identification of frailty risk among stroke patients and prompt clinical intervention targeting associated risk factors are of critical importance. In recent years, the number of published frailty risk prediction models for stroke patients has increased, with each model incorporating different predictor variables. However, the methodological quality, predictive accuracy, and generalizability of these models remain unclear, and no comprehensive systematic review or meta-analysis has been conducted on this topic. Thus, this study aims to perform a systematic review and meta-analysis of existing frailty risk prediction models for stroke patients in order to provide evidence-based insights for clinical decision-making.

## 2 Aims

To systematically review the frailty risk prediction models for stroke patients.

## 3 Methods

The protocol for this study is registered with PROSPERO (registration number: CRD420251075547).

### 3.1 Search methods

**Figure 1:**
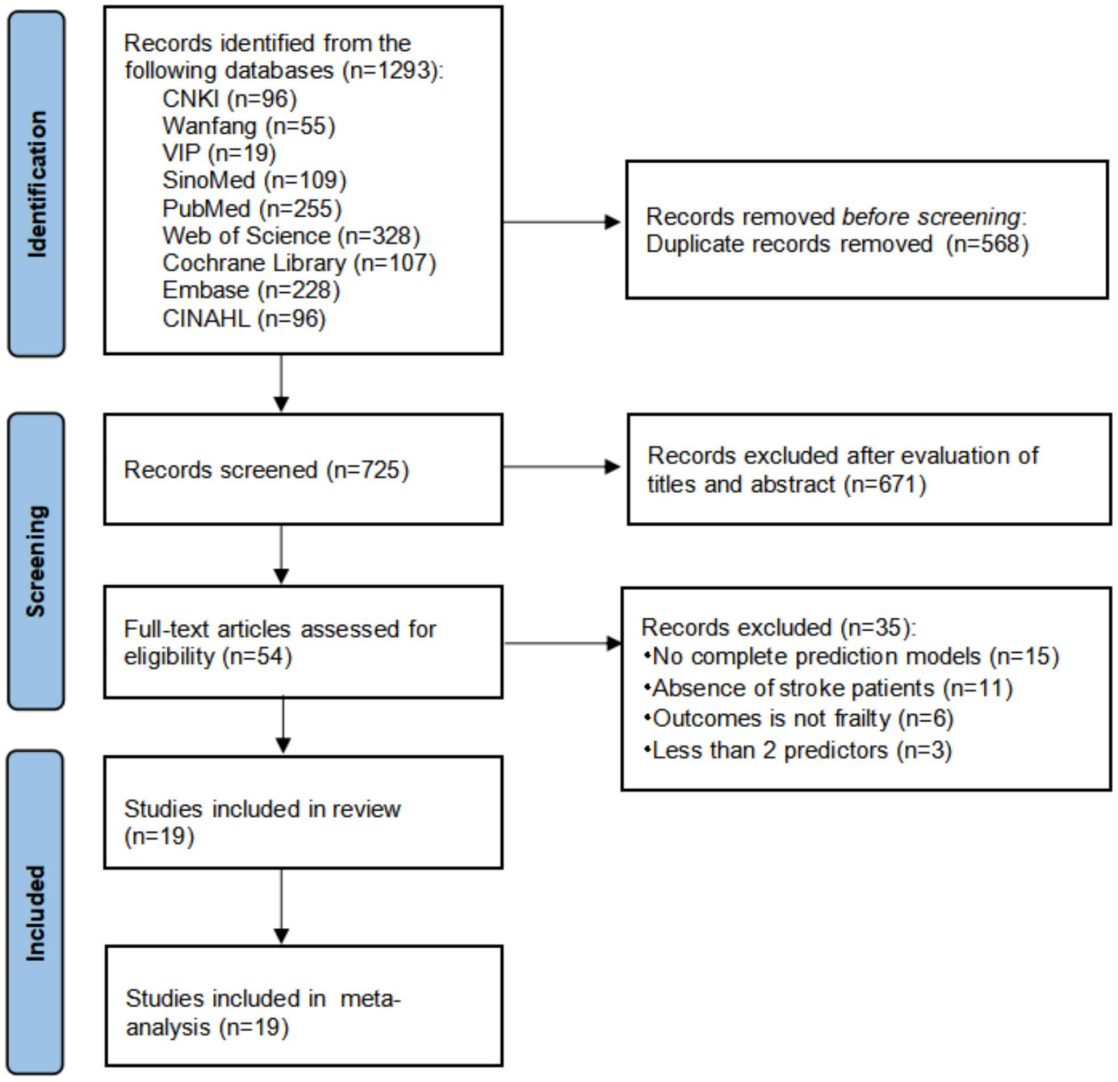
Flowchart of the literature search and selection based on preferred reporting items for systematic reviews and meta-analyses (PRISMA) 2020.

A comprehensive search was conducted on PubMed, Web of Science, the Cochrane Library, Embase, CINAHL, China National Knowledge Infrastructure (CNKI), Wanfang Database, China Science and Technology Journal Database (VIP) and SinoMed from the inception of the databases to May 31, 2025 to identify relevant studies. We used a combination of Medical Subject Headings (MeSH) and free-text terms: stroke, acute stroke, cerebrovascular disease, cerebrovascular accident, cerebral embolism, cerebral thrombosis, cerebral infarction, ischemic stroke, haemorrhagic stroke, cerebral haemorrhage, frailty, frailty syndrome, frail, fragile, risk prediction model, risk prediction, prediction model, risk assessment, risk calculation, risk factor, predictor, model and risk score. Furthermore, we manually reviewed the reference lists of the included studies to identify additional potentially relevant studies. The detailed search strategies in Pubmed are shown in Figure 1.

### 3.2 Inclusion and exclusion criteria

For the systematic review, we utilized the PICOTS system, as recommended by the Checklist for Critical Appraisal and Data Extraction for Systematic Reviews of Prediction Modelling Studies (CHARMS) (17), to provided a comprehensive description of the study population, intervention, comparator, outcome, timing, and setting in this review. The key items of this systematic review were described below.

P (Population): Patients with stroke.

I (Intervention model): Risk prediction models for frailty in stroke patients that were developed and published (predictors ≥ 2).

C (Comparator): No competing models.

O (Outcome): The outcome was frailty status (for cross-sectional studies) or frailty incidence (for prospective studies).

T (Timing): No limitations.

S (Setting): Inpatient and (or) outpatient settings.

The inclusion criteria for studies were: (1) patients were diagnosed with stroke, including ischemic stroke and hemorrhagic stroke; (2) the studies focused on the development and (or) validation of risk prediction models for the occurrence of frailty in stroke patients; (3) the types of studies included cross-sectional studies, cohort studies, and case–control studies; (4) Outcome definition: Cross-sectional studies assessed frailty status (presence or absence at the time of evaluation); prospective studies assessed frailty incidence (new cases during follow-up). All studies used validated frailty instruments. Cross-sectional models evaluate concurrent discrimination, not prospective risk prediction.

The exclusion criteria for studies were: (1) studies only explored the risk factors influencing the occurrence of frailty in stroke patients without developing prediction models; (2) systematic reviews, meta analyses and conference abstracts, etc.; (3) the full text was unavailable or data information was incomplete; (4) duplicate publications.

### 3.3 Study selection

Studies were screened independently by two researchers with systematic evidence-based training. First, duplicate studies were eliminated. Second, two researchers independently read the titles and abstracts of the studies and excluded studies that clearly did not fit the topic of the study. Finally, two researchers reviewed the full text to determine the final number of studies to be included based on inclusion and exclusion criteria. If there was disagreement between the parties on study selection, consensus was reached after discussion with the third researcher.

### 3.4 Data extraction

Relevant data was extracted based on the final included studies. The data extraction included three parts: (1) basic information of the studies: author, year of publication, country, data source, study design, outcome measures, frailty cases/sample size, candidate predictors and model presentation. (2) Basic characteristics of the models: missing data, continuous variable, variable selection, events per variable, modeling method, validation method, candidate variables, modeling method, model validation method, area under the curve, and calibration method. (3) Predictors of the models: the number of predictors and specific predictors. Any disagreement was settled by consensus.

### 3.5 Quality appraisal

Two researchers independently assessed the risk of bias and applicability of the included studies with the Prediction Model Risk of Bias Assessment Tool (PROBAST) (18). The PROBAST assessment comprises 20 questions categorized into four domains: participants, predictors, outcomes, and analysis. Each question can be answered as “yes,” “probably yes,” “no,” “probably no” or “no information”. If all four domains are rated as low, the overall bias risk can be considered low; if the bias risk was high in any one domain, the overall bias risk is considered high; if the bias risk was unclear in any one domain and low in other domains, the overall bias risk is considered unclear.

### 3.6 Synthesis

Stata software (version18.0) was utilized for the meta-analysis. Heterogeneity was examined using the I^2^ index and the Cochrane Q test. The *I*^2^ index provides a measure of heterogeneity with values of 25, 50, and 75%, indicating low, moderate, and high heterogeneity. If *I*^2^ > 50%, the random effects model was used, and if not, the fixed effects model was used. Count data were expressed as Odd Ratio (OR) and 95% Confidence Interval (CI). Subgroup analysis and sensitivity analysis were performed for studies with significant clinical heterogeneity. Egger’s test was used to analyze publication bias. Because AUC is bounded (0.5–1), all values were logit-transformed prior to pooling: logit (AUC) = ln [AUC/(1 − AUC)]. For studies not reporting standard error (SE), it was calculated from the 95% CI using SE = (CI_upper_ − CI_lower_)/3.92, then converted to logit scale as SE_logit = SE/[AUC × (1 − AUC)]. Pooled estimates were obtained using a random-effects inverse variance model (DerSimonian–Laird) and back-transformed to original AUC scale. Correlated AUC estimates from studies developing multiple models were treated as independent; this limitation is acknowledged.

## 4 Results

### 4.1 Search results

Figure 1 shows the literature screening process according to the PRISMA 2020 flowchart (19). We searched a total of 1,293 records from the comprehensive database, and 568 duplicate records were removed. After reading the titles and abstracts, 671 irrelevant records were excluded. By reading the full text of the articles, 35 articles were excluded (15 articles did not develop risk prediction models, the populations of 11 articles were not patients with stroke, the outcomes of 6 articles were not frailty status or frailty incidence, and 3 articles had fewer than 2 predictors). 19 studies were finally included (20–38). Among them, one study developed two models using logistic regression and artificial neural networks (33), one study developed two models using the random forest methods and logistic regression (34), and one study developed four models through logistic regression and the eXtreme gradient boosting (XGBoost) method (37). Eventually, a total of 24 risk predictive models for frailty in stroke patients were included.

### 4.2 Basic characteristics of the included studies

Table 1 summarized the basic characteristics of the included studies. A total of 19 studies, all from China, were published between 2023 to 2025, and two of which were published in English. Of the included studies, six were retrospective cohort studies, eight were prospective cohort studies, four were cross-sectional studies, and one was nested case–control study, including one multi-center study. In terms of the study population, all were patients with ischemic stroke or cerebral infarction. Among the frailty assessment tools implemented in stroke patient cohorts, five studies employed the Fried Frailty Phenotype; seven studies utilized the Frail scale, encompassing five critical domains: fatigue, resistance, ambulation, illness, and weight loss; five studies incorporated the Tilburg Frailty Indicator; one study leveraged the Edmonton Frailty Scale; and one study adopted the Elderly Frailty Assessment Scale. Furthermore, nine studies developed predictive models presented in the form of a nomogram.

**Table 1:** Basic characteristics of the included studies (*n* = 19).

Author (year)	Country	Study population	Study design	Data source	Outcome measures	Frailty cases/sample size (%)	Candidate predictors	Model presentation
Chen et al. (2023) (20)	China	Acute ischemic stroke	Retrospective cohort study	Ninety-sixth Hospital of the People’s Liberation Army Joint Logistics and Security Forces (Single-center)	Fried Frailty Phenotype	57/156 (36.54%)	14	—
Ding et al. (2023) (21)	China	Stroke	Retrospective cohort study	Affiliated Hospital of Jiangxi University of Traditional Chinese Medicine (Single-center)	Frail scale	83/132 (62.88%)	12	Nomogram
Gao et al. (2025) (22)	China	Ischemic stroke	Prospective cohort study	Department of Neurology, a tertiary hospital in Shandong Province, China (Single-center)	Frail scale	196/331 (59.21%)	21	Nomogram
Jiang et al. (2024) (23)	China	Ischemic stroke	Prospective cohort study	Department of Emergency Medicine, The First Affiliated Hospital of Bengbu Medical University, Bengbu, China (Single-center)	Frail scale	109/231 (47.19%)	22	—
Ma et al. (2025) (38)	China	Ischemic stroke	Prospective cohort study	Neurological Diagnosis and Treatment Center of the Second Affiliated Hospital of Xinjiang Medical University (Single-center)	Frail scale	61/322 (18.94%)	12	Random forest model
Qiao et al. (2025) (37)	China	Ischemic stroke	Prospective cohort study	Affiliated Hospital of North China University of Science and Technology (Single-center)	Fried frailty phenotype	375/904 (41.48%)	10	Bayesian network model
Wang et al. (2024) (25)	China	Acute strike	Prospective cohort study	A tertiary hospital in Suzhou, China (Single-center)	Tilburg frailty indicator	70/193 (36.27%)	20	Nomogram
Zhang et al. (2024) (26)	China	Ischemic stroke	Prospective cohort study	Department of Neurology, a tertiary hospital in Qingdao, China (Single-center)	Tilburg frailty indicator	134/441 (30.39%)	19	Decision tree
Gao (2024) (27)	China	Acute cerebral infarction	Retrospective cohort study	Department of Geriatric Neurology, The First Affiliated Hospital of Henan University (Single-center)	Frail scale	209/311 (67.20%)	16	Nomogram
Chen (2024) (28)	China	Stroke	Cross-sectional study	A tertiary hospital in Jingzhou (Single-center)	Tilburg frailty indicator	225/319 (70.53%)	43	Decision tree model
Shen et al. (2024) (29)	China	Ischemic stroke	Cross-sectional study	Neurology, a medical university hospital(Single-center)	Tilburg frailty indicator	263/452 (58.19%)	42	Nomogram
Wang et al. (2024) (30)	China	Cerebral infarction	Prospective cohort study	Hangzhou Third people’s Hospital (Single-center)	Edmonton frailty scale	70/146 (47.95%)	23	—
Zhong et al. (2023) (31)	China	Acute cerebral infarction	Retrospective cohort study	Suzhou Hospital of Integrative Medicine (Single-center)	Fried frailty phenotype	125/195 (64.10%)	18	Nomogram
Liang et al. (2024) (32)	China	Cerebral infarction	Retrospective cohort study	Department of Neurology, a city tertiary care hospital(Single-center)	Frail scale	28/110 (25.45%)	17	Nomogram
Zhang et al. (2023) (33)	China	Stroke	Prospective cohort study	A hospital in Jinzhou (Single-center)	Elderly frailty assessment scale	249/532 (46.80%)	18	Nomogram, neural network model
Zhang et al. (2025) (34)	China	Stroke	Retrospective study	Dongping Hospital of Shandong First Medical University (Single-center)	Fried Frailty Phenotype	98/265 (36.98%)	20	Random forest model
Zhang (2024) (35)	China	Acute ischemic stroke	Cross-sectional study	Department of Neurology, a tertiary hospital in Zhengzhou (Single-center)	Frail scale	122/395 (30.89%)	27	Nomogram
Li (2024) (36)	China	Stroke	Cross-sectional study	Department of Neurology, 2 tertiary hospitals in Gansu Province (multi-center)	Tilburg frailty indicator	306/432 (70.83%)	24	Decision tree model
Qiao et al. (2025) (37)	China	Ischemic stroke	Nested case–control study	Affiliated Hospital of North China University of Science and Technology (single-center)	Fried frailty phenotype	220/826 (26.63%)	15	—

“—”, Not report.

### 4.3 Basic characteristics of model development and validation

Table 2 described the basic characteristic of the frailty risk prediction models for stroke patients. In terms of modeling method, 15 models (20–25, 27, 29–35, 37) utilized logistic regression, while the remaining models employed decision trees, random forests, Bayesian networks, neural networks, and other machine learning algorithms, respectively. Regarding model validation, a total of 3 model (29, 31, 33) underwent both internal and external validation, with 3 models (26, 29, 31) underwent external validation and 15 models (20–22, 25–29, 31, 33–38) underwent internal validation. The internal validation procedures included methods such as Bootstrap, five-fold cross-validation, and ten-fold cross-validation. Concerning the selection of candidate variables, 15 studies conducted univariate analysis followed by multivariate analysis, the rest of the studies used feature importance screening based on random forest model and Least Absolute Shrinkage and Selection Operator (LASSO) regression method. Regarding variable handling, 4 studies (21–23, 25) partially converted continuous variables into categorical variables, while another 6 studies (24, 28, 31–33, 37) fully transformed all continuous variables into categorical variables. In terms of missing data, one study (35) reported no missing data, 4 studies (26, 30, 33, 36) explicitly stated that missing cases were excluded, and the remainder did not provide information regarding the presence or handling of missing data.

**Table 2:** Basic characteristics of risk prediction models for frailty in stroke patients (*n* = 19).

Author (year)	Missing data	Continuous variable	Variable selection	EPV	Modeling method	Validation method	AUC (model development/model validation)	Calibration method
Chen et al. (2023) (20)	Not report	Continuous variables	Univariate and multivariate analysis	14.25	LR	Internal validation	0.840/0.676	H-L test, *p* = 0.072
Ding et al. (2023) (21)	Not report	Continuous variables Categorical variables	Univariate and multivariate analysis	9.8	LR	Internal validation	—/0.928	H-L test, *p* = 0.522
Gao et al. (2025) (22)	Not report	Continuous variables Categorical variables	Univariate and multivariate analysis	22.5	LR	Bootstrap	0.937/0.937	Calibration curve, DCA
Jiang et al. (2024) (23)	Not report	Continuous variables Categorical variables	Univariate and multivariate analysis	18.17	LR	—	0.780/—	H-L test, *p*>0.05
Ma et al. (2025) (38)	Not report	Continuous variables	—	12.2	RF	Bootstrap	0.795/0.816	—
Qiao et al. (2025) (24)	Not report	Categorical variables	Univariate and multivariate analysis	47	LR, BN	—	—	—
Wang et al. (2024) (25)	Not report	Continuous variables Categorical variables	Univariate and multivariate analysis	17.5	LR	Bootstrap	—/0.839	H-L test, *p* = 0.139
Zhang et al. (2024) (26)	Excluded	Continuous variables	Univariate and multivariate analysis	26.8	DT	Five-fold cross validation	0.94/0.92	—
Gao (2024) (27)	Not report	Continuous variables	Univariate and multivariate analysis	11.67	LR	Internal validation	0.902/0.931	H-L test, *p* = 0.724,calibration curve
Chen (2024) (28)	Not report	Categorical variables	CART	—	DT	Ten-fold cross validation	—	—
Shen et al. (2024) (29)	Not report	Continuous variables	Univariable analysis, LASSO regression, RF-RFE	21	LR	Bootstrap, External validation	0.904/0.920	H-L test, *p* = 0.210, DCA, calibration curve, Brier score
Wang et al. (2024) (30)	Excluded	Continuous variables	Univariate and multivariate analysis	35	LR	—	0.799/—	—
Zhong et al. (2023) (31)	Not report	Categorical variables	Univariate and multivariate analysis	4.27	LR	Internal validation, external validation	0. 853/0. 844	DCA, calibration curve
Liang et al. (2024) (32)	Not report	Categorical variables	Univariate and multivariate analysis	14	LR	—	0.825/—	H-L test, *p* = 0.754,calibration curve
Zhang et al. (2023) (33)	Excluded	Categorical variables	Univariate and multivariate analysis	29.33	LR, ANN	Internal validation, external validation	LR: 0.908/0.923 ANN: 0.904/0.916	—
Zhang et al. (2025) (34)	Not report	Continuous variables	Univariate and multivariate analysis	14	RF, LR	five-fold cross validation	RF: —/0.728 LR: —/0.629	—
Zhang (2024) (35)	No missing data	Continuous variables	Univariate and multivariate analysis	20.33	LR	Internal validation	—/0.812	H-L test, *p* = 0.427
Li (36)	Excluded	Continuous variables	CART	42	DT	Internal validation, confusion matrix	—/0.854	—
Qiao et al. (2025) (37)	Not report	Categorical variables	Univariate and multivariate analysis	31.43	LR, XGBoost	five-fold cross validation	Classical prediction model:0.767; Classical prediction model + RI:0.787; Classical prediction model+PNI:0.774; Classical prediction model + PNI + RI:0.807	H-L test, *p*>0.05,calibration curve

“—”, Not report; LR, Logistic regression; RF, Random forest; BN, Bayesian network; DT, Decision tree; ANN, Artificial neural network; EPV, Events per variable; AUC, Area under the curve; DCA, Decision curve analysis; H-L test, Hosmer-Lemeshow test; LASSO, Least Absolute Shrinkage and Selection Operator; CART, Classification And Regression Tree; RF-RFE, random forest recursive feature elimination; XGBoost, extreme gradient boosting; PNI, Prognostic nutritional index; RI, Vertebral artery resistance index.

24 models reported AUC values ranging from 0.629 to 0.940 during model development. Among these, twenty models described AUC values between 0.629 and 0.940 during internal validation, while 4 models presented AUC values from 0.844 to 0.923 in external validation. Only two models demonstrated an AUC < 0.7 (20, 34), all other AUCs were > 0.7. Calibration was assessed using various methods, including the Hosmer-Lemeshow test (*n* = 9), calibration curves (*n* = 6), decision curves (*n* = 3), and the Brier score (*n* = 1). Detailed results are presented in Table 2.

The number of predictors and the content of specific predictors for each model are given in Table 3. Of these, 24 models contained a total of 104 risk predictor variables. The three most commonly reported predictors were age (*n* = 13), activities of daily living (*n* = 6), and NIHSS score (*n* = 5).

**Table 3:** Specific predictors of the frailty risk prediction model for stroke patients (*n* = 19).

Author (year)	Number of predictors	Specific predictors
Chen et al. (2023) (20)	4	Age, diabetes, massive cerebral infarction, dysphagia
Ding et al. (2023) (21)	5	Age, diabetes, malnutrition, admission NIHSS score, depression
Gao et al. (2025) (22)	6	Number of strokes, presence or absence of movement disorders, multimorbidity, mRS score, MNA-SF score, ADL score
Jiang et al. (2024) (23)	6	Age, Physical Activity, Nutritional Risk, ADL, Degree of Neurologic Impairment, B-APQ Score
Ma et al. (2025) (38)	5	Type of disease, sleep, 36-item Brief Health Questionnaire score, Activities of Daily Living Scale score, personal monthly income(<2000yuan)
Qiao et al. (2025) (24)	6	Number of cerebrovascular disease episodes, physical exercise, depression, self-care ability, PNI, hypertension
Wang et al. (2024) (25)	4	Age, history of diabetes, PHQ-9 score and Braden score
Zhang et al. (2024) (26)	5	NIHSS scores, geriatric depression, grip strength, social support, and general self-efficacy
Gao (2024) (27)	6	Age, NHISS score, head and neck arterial stenosis, head and neck arterial occlusion, hyperhomocysteine, activities of daily living
Chen (2024) (28)	4	Age, multiple chronic conditions, ability to perform activities of daily living, social support
Shen et al. (2024) (29)	9	Marital Status, Smoking, Physical Activity, History of Falls in Previous Year, Polypharmacy, Albumin Levels, Dysphagia, Cognitive Impairment
Wang et al. (2024) (30)	2	Hemoglobin, homocysteine (Hcy)
Zhong et al. (2023) (31)	11	Age >75 years, dysphagia duration ≥21 d, living alone, self-paying, poor economic status, ≥3 comorbidities, ≥3 medications, physical dyskinesia, depression, malnutrition, low level of social support
Liang et al. (2024) (32)	2	Anxiety, age
Zhang et al. (2023) (33)	6	Age ≥80 years, sleep disturbance, impaired instrumental activities of daily living, history of falls, living alone, physical activity
Zhang et al. (2025) (34)	7	Baseline NIHSS score, MoCA score, age, albumin, qEEG delta wave power, TMS cortical resting phase, GDS-15 score
Zhang (2024) (35)	6	Age, BMI, home residence, HDL, Charlson’ syndrome index, EQ-5D-3L
Li (2024) (36)	3	Age, NIHSS score, mode of residence
Qiao et al. (2025) (37)	7	Age >70 years old, PNI ≤ 46.3, comorbidity ≥2, moderate to severe cerebral white matter lesions, RI > 0.67, stroke onset time≥2, moderate and high physical activity levels

NIHSS, the National Institutes of Health stroke scale; mRS, modified rankin scale; MNA-SF, mini-nutritional assessment short-form; ADL, activities of daily living; B-APQ, the Brief Ageing Perceptions Questionnaire; PHQ-9, patient health questionnaire; MoCA, Montreal Cognitive Assessment; qEEG, quantitative electroencephalography; TMS, transcranial magnetic stimulation; GDS-15, Geriatric Depression Scale; BMI, Body mass index; HDL, High Density Lipoprotein; EQ-5D-3L, Three-level EuroQol Five-Dimensional Questionnaire; PNI, Prognostic Nutrition Index; RI, resistance index.

### 4.4 Result of quality assessment

Table 4 summarizes the risk of bias and applicability of the included studies. In the risk of bias assessment, all 19 studies exhibited a high risk of bias in at least one domain. Specifically, in the participants domain, ten studies were considered to be at a high risk of bias primarily due to the use of inappropriate data source, including cross-sectional and retrospective studies (20, 21, 27–29, 31, 32, 34–36). In the predictors domain, 7 studies exhibited an unclear risk of bias, as they did not report the quality control measures employed for predictor assessment, which may be attributable to their retrospective design (20, 21, 23, 27, 31, 34, 38). In the outcome domain, 14 studies the risk of bias was rated as unclear, 14 studies did not report the time interval between predictor assessment and outcome determination (20, 21, 23, 24, 27–29, 31–36, 38), one study did not provide blind assessment between the outcome and predictors (24).

**Table 4:** Risk of bias and applicability evaluation of the models (*n* = 19).

Author (year)	Risk of bias				Applicability			Overall	
	Participants	Predictors	Outcome	Analysis	Participants	Predictors	Outcome	Risk of bias	Applicability
Chen et al. (2023) (20)	**−**	**?**	**?**	**−**	**+**	**+**	**+**	**−**	**+**
Ding et al. (2023) (21)	**−**	**?**	**?**	**−**	**+**	**+**	**+**	**−**	**+**
Gao et al. (2025) (22)	**+**	**+**	**+**	**−**	**+**	**+**	**+**	**−**	**+**
Jiang et al. (2024) (23)	**+**	**?**	**?**	**−**	**+**	**+**	**+**	**−**	**+**
Ma et al. (2025) (38)	**+**	**?**	**?**	**−**	**−**	**+**	**+**	**−**	**−**
Qiao et al. (2025) (24)	**+**	**+**	**?**	**−**	**+**	**+**	**+**	**−**	**+**
Wang et al. (2024) (25)	**+**	**+**	**+**	**−**	**−**	**+**	**+**	**−**	**−**
Zhang et al. (2024) (26)	**+**	**+**	**+**	**−**	**+**	**+**	**+**	**−**	**+**
Gao (2024) (27)	**−**	**?**	**?**	**−**	**+**	**+**	**+**	**−**	**+**
Chen (2024) (28)	**−**	**+**	**?**	**−**	**+**	**+**	**+**	**−**	**+**
Shen et al. (2024) (31)	**−**	**+**	**?**	**−**	**+**	**+**	**+**	**−**	**+**
Wang et al. (2024) (30)	**+**	**+**	**+**	**−**	**+**	**+**	**+**	**−**	**+**
Zhong et al. (2023) (31)	**−**	**?**	**?**	**−**	**+**	**+**	**+**	**−**	**+**
Liang et al. (2024) (32)	**−**	**+**	**?**	**−**	**+**	**+**	**−**	**−**	**−**
Zhang et al. (2023) (33)	**+**	**+**	**?**	**−**	**+**	**+**	**+**	**−**	**+**
Zhang et al. (2025) (34)	**−**	**?**	**?**	**−**	**−**	**+**	**+**	**−**	**−**
Zhang (2024) (35)	**−**	**+**	**?**	**−**	**−**	**+**	**+**	**−**	**−**
Li (2024) (36)	**−**	**+**	**?**	**−**	**+**	**+**	**+**	**−**	**+**
Qiao et al. (2025) (37)	**+**	**+**	**+**	**−**	**−**	**+**	**+**	**−**	**−**

+, low risk of bias; −, high risk of bias;?, unclear risk of bias.

In the analysis domain, all 19 studies were found to have high risk of bias. Among them, 9 studies met the recommended the number of events per variable (EPV) of ≥ 20 events per candidate variable (22, 24, 26, 29, 30, 33, 35–37). 10 studies partially or completely converted continuous variables into categorical variables without reporting solid standards (21–25, 28, 31–33, 37). 4 studies did not include all enrolled participants in the analysis, 4 studies directly excluded missing data and did not handle it appropriately (26, 30, 33, 36), 1 study reported no missing data (35) and others did not report information on missing data. Only 3 studies avoid selecting variables based on univariate analysis (28, 36, 38). 4 studies comprehensively assess the discrimination and calibration of prediction models (27, 29, 32, 37). 4 studies did not account for model overfitting, underfitting and optimism in model performance because they did not report internal validation (23, 24, 30, 32). 3 studies did not report the coefficients of final predictors in the regression model (28, 36, 38). Complexities in the data were not reported in any included studies.

In terms of the assessment of applicability risk, 6 studies were rated as high risk, while 13 studies had a low risk. In the participants domain, 5 studies were considered at high risk of applicability. Among these, 2 studies were limited by the inclusion of participants from a specific stroke subgroup (37, 38), while the other three studies focused exclusively on first-onset stroke with symptom onset within seven days (25, 34, 35). In the predictors domain, all included studies had low risk. In the outcome domain, one study was at high risk due to including prefrailty into the frailty group (32).

### 4.5 Meta-analysis of validation models

**Figure 2:**
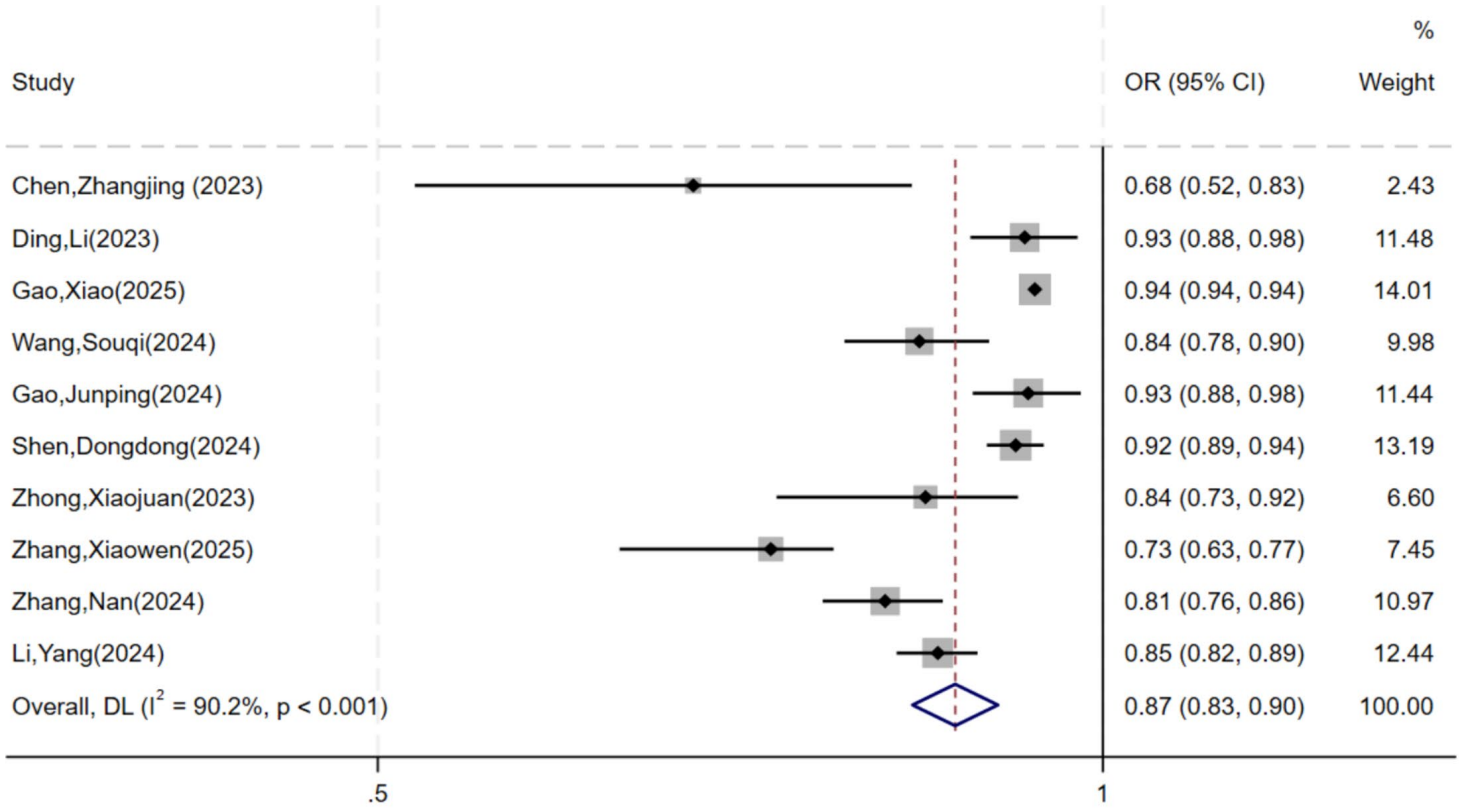
Forest plot of the random effects meta-analysis of pooled AUC estimates for 10 validation models.

Ten studies were included in the meta-analysis due to insufficient reporting on the development details of models. The pooled AUC using random-effects model was 0.87 (95% CI: 0.83–0.90) (Figure 2), with extreme heterogeneity (*I*^2^ = 90.2%, *p* < 0.001). Given this extreme heterogeneity and the substantial differences across studies in frailty definitions, model types, and designs, this pooled estimate should not be interpreted as reflecting a single underlying effect. Rather, it serves as a descriptive summary of reported discrimination values in the current literature. Pooling was retained to provide a quantitative overview, while acknowledging its exploratory nature and limitations. Narrative synthesis may be more appropriate in future updates as more studies become available. All studies were at high risk of bias, further suggesting the estimate is likely inflated. Egger’s test (*p* > 0.05) showed that there was no significant publication bias.

### 4.6 Subgroup and sensitivity analyses

**Figure 3:**
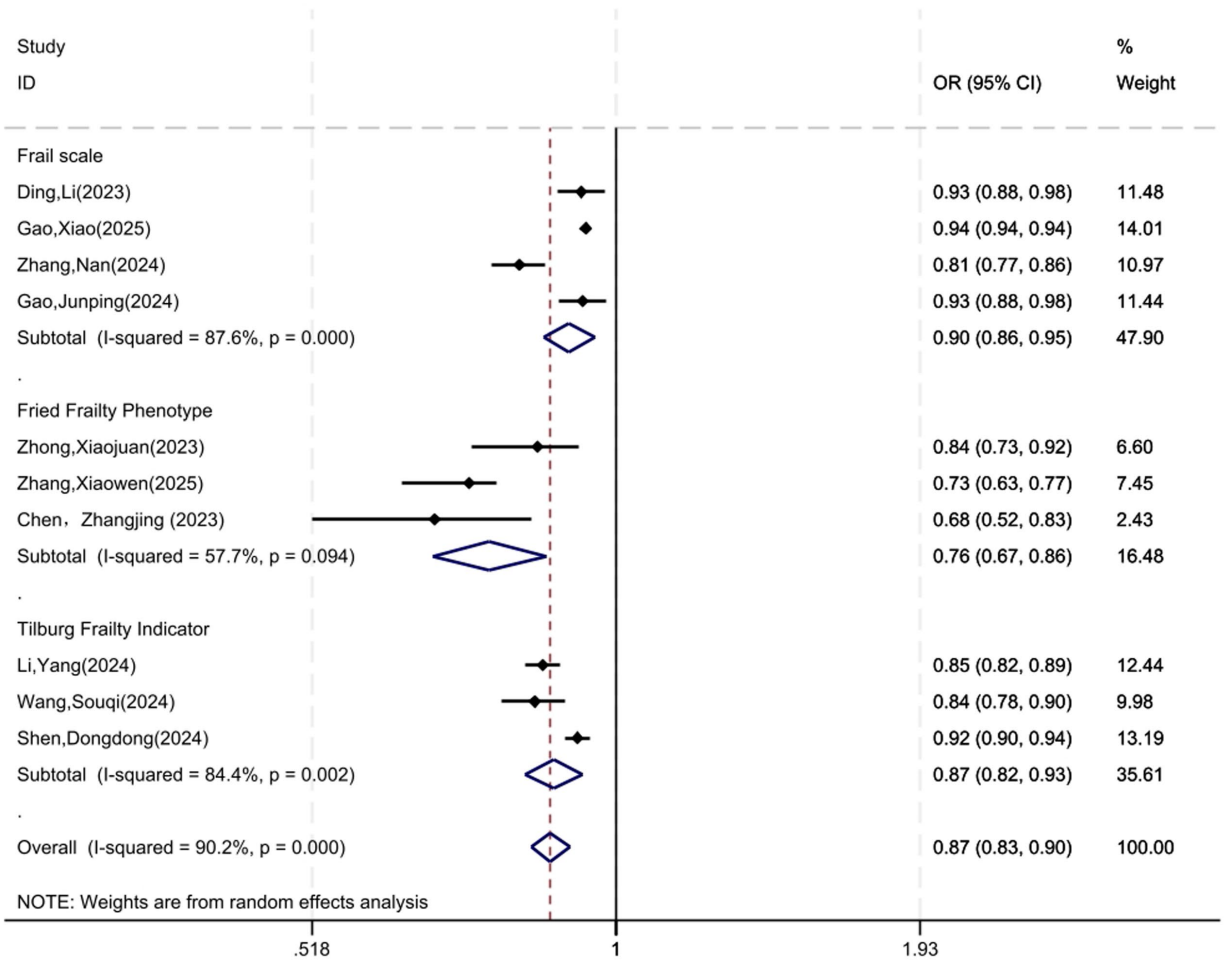
Forest plot for subgroup analysis by frailty instrument.

Frailty instrument type was a major source of heterogeneity, with the Fried Phenotype showing lower predictive performance in stroke patients. The results of subgroup analyses are shown in Figure 3.

Excluding the study with an extremely narrow CI (22) yielded a pooled AUC of 0.86 (95% CI: 0.82–0.90) with reduced but still substantial heterogeneity (*I*^2^ = 83.5%), indicating the overall estimate was robust. After excluding three cross-sectional studies, the pooled AUC of the remaining seven studies was 0.87 (95% CI: 0.82–0.92), and the heterogeneity was still high (*I*^2^ = 86.3%). This result aligns with the main analysis (0.87), but the cross-sectional models reflect discrimination for current frailty status rather than true predictive validity. Their inclusion does not materially affect the summary estimate but conceptually differs from prospective risk prediction.

## 5 Discussion

Frailty is a syndrome that can be prevented through early identification and intervention. Risk assessment is regarded as a critical strategy for preventing frailty (39). A comprehensive evaluation of the potential risk of post-stroke frailty facilitates early identification and timely intervention, thereby helping to reduce adverse outcomes among stroke patients (40). Therefore, in recent years, the development of frailty risk prediction models for stroke patients has increasingly garnered attention due to its clinical significance. In this systematic review, we identified 19 studies reporting 24 frailty risk prediction models for stroke patients, all developed between 2023 and 2025. We also summarized the characteristics and performance of these models while assessing their bias risk and applicability. These findings will provide reference value for the construction, validation, update and application of frailty models for stroke patients in the future.

The prediction performance of the included models was primarily assessed in terms of model discrimination and calibration. Additionally, sensitivity, specificity, and accuracy were partially evaluated to further validate the models’ effectiveness. Discrimination refers to the ability of a model to accurately distinguish between individuals with different levels of future disease risk, which is commonly expressed as AUC or CI. This systematic review found that, except for two models with AUCs of 0.676 and 0.629 (20, 34), all other models had an AUC ≥ 0.7. However, given that all included studies were at high risk of bias due to small samples, single-center designs, retrospective or cross-sectional data, inadequate handling of missing data, and categorization of continuous predictors, this apparent performance is likely inflated by overfitting and optimism bias. Therefore, the pooled estimate should be viewed as an exploratory summary of current literature, not as reliable evidence of predictive accuracy. The calibration degree reflects the consistency between the predicted risk and the actual risk, which is commonly evaluated by the Hosmer-Lemeshow test (41). A smaller Hosmer-Lemeshow test statistic (and thus a larger *p*-value) indicates better model calibration. However, a non-significant H-L test alone does not confirm good calibration, as the test has low statistical power in small samples and may fail to detect miscalibration. More robust measures such as calibration plots, calibration slope, and Brier score were reported in only a few studies (29). Future research should prioritize these metrics over sole reliance on H-L *p*-values to comprehensively evaluate calibration. In addition, during the model evaluation, we observed inadequate reporting adherence to the Transparent Reporting of a Multivariate Prediction Model for individual Prognosis (TRIPOD) statement in several studies, with insufficient methodological details such as sample-size calculation, handling of missing data, and model presentation, highlighting the need for adherence to the TRIPOD guidelines in future research. In conclusion, all studies were at high risk of bias according to PROBAST, which limits the practical application of these prediction models. Future studies should prioritize the development of new models with larger sample sizes, rigorous study designs, multicenter external validation, and better reporting transparency.

In terms of data sources, the majority of the included studies were single-center and characterized by relatively small sample sizes. Only one study was from multiple centers (36), while the remaining studies were all based on single-center designs. Among the included studies, 6 studies were retrospective study, 4 were cross-sectional study, 8 were prospective cohort study, and 1 was nested case–control study. Retrospective studies have the problem of recall bias (42), and cross-sectional studies have only preliminarily determined the association between influencing factors and frailty. Cross-sectional models included in this review assess the ability to discriminate current frailty status, not predict future onset. These are effectively diagnostic classification models, not prognostic prediction models. Because predictors and outcome were measured simultaneously, these models cannot establish temporal ordering and may overestimate predictive performance. Including them alongside prospective prediction models further complicates the interpretation of pooled discrimination estimates. Although sensitivity analysis showed minimal impact on the pooled estimate, this conceptual distinction should be considered when interpreting findings. Prospective cohort studies should be considered in the future, which can better reveal the causal relationship between influencing factors and frailty and reduce the risk of bias (43). The prevalence of frailty varied significantly in the included studies, ranging from 18.94 to 70.53%. This variation may be attributed to differences in study settings, participants’ characteristics, and frailty assessment tools. A variety of standardized instruments are currently available for assessing frailty in clinical practice and research. This review revealed several frailty tools for assessing the outcomes of frailty risk prediction models for stroke patients. The tools used to assess frailty in stroke patients included Frail scale, Fried Frailty Phenotype, Tilburg Frailty Indicator, Elderly frailty assessment scale and Edmonton scale. Among these, the Fried Frailty Phenotype and the Frail scale were the two frequently utilized tools. This systematic review found that the heterogeneity of frailty assessment tools was an important reason for the differences in the performance of various prediction models. Subgroup analysis by frailty instrument revealed marked differences in pooled AUC: Fried phenotype (0.76), Frail scale (0.90), Tilburg Indicator (0.87). These instruments measure distinct frailty constructs—Fried focuses on physical phenotype, while Frail scale and Tilburg incorporate multidimensional (physical, psychological, social) domains. This conceptual heterogeneity limits direct comparability of predictive performance across models, as each tool defines the outcome differently. The lower performance of Fried phenotype may reflect overlap between physical frailty criteria and stroke-related neurological deficits, whereas multidimensional tools capture broader aspects of post-stroke vulnerability. Although subgroup analysis by instrument is appropriate, residual differences in construct measurement should be considered when interpreting pooled estimates. In the future, frailty assessment tools can be selected according to the different research objects, and multi-center, large-sample prospective studies can be carried out to reduce the risk of bias.

In the analysis field, the sample size of the 9 studies was insufficient, which may lead to over-fitting of the prediction model and affect clinical decision-making. It is suggested that large samples from multiple sources should be used in future studies. For logistic regression models, EPV was calculated as events per candidate predictor. For machine learning models, EPV is not directly applicable; we report the number of events and candidate predictors for transparency, but this metric should be interpreted with caution for non-regression methods. It is suggested that the EPV for each independent variable should be ≥ 20 and the sample size for validation should be ≥ 100 in model development (44). Fourteen studies did not report the handling methods for missing data, and four studies directly deleted the missing data. This would lead to a bias in the correlation between predictors and outcomes, thereby affecting the model performance. It is recommended to use multiple interpolation methods to handle missing data. The multiple imputation method can effectively reduce the adverse effect of missing data on the performance of the model and ensure the reliability of the results (45). Only one study used Lasso regression method to screen predictors (29), and most studies used univariate and multivariate analysis, which could easily cause omission of important predictors and bias. Therefore, it is recommended that future research adopt a more comprehensive strategy for predictor selection based on univariate analysis results, such as literature reviews, expert consultations, stepwise forward or backward selection, or Lasso regression (46). Ten studies compromised variable continuity by converting all or part of continuous variables into categorical variables. It is recommended that researchers retain the original values in the model to minimize the risk of introducing bias (47). Five studies relied solely on the H-L test for calibration assessment. To comprehensively evaluate the predictive performance of prediction models, it is advised that researchers assess both discrimination and calibration. This evaluation should include complementary statistical measures such as the Brier score and calibration intercept, in conjunction with the Hosmer-Lemeshow test. This review found that most models were developed without any form of validation, particularly external validation. Validation is an important step to promote the model outwards (48). Only three models were externally validated in this systematic review, and the study populations were all from China, which may limit the prediction effect of the models in other countries, regions and populations, thereby limiting the extrapolation and generalization of the models. External validation is used to evaluate the stability and validity of the model, which saves time and economic cost compared with reconstructing the model (49). Therefore, future studies should prioritize multi-center, international collaborations to develop and validate frailty prediction models across diverse populations. External validation in different geographic regions and healthcare settings is essential before any model can be considered for clinical implementation outside China. At present, Logistic regression modeling is widely used in the included studies, but this traditional modeling method has problems such as missing data and nonlinear relationships, which leads to poor model performance. Compared with traditional modeling methods, machine learning methods such as random forests, neural networks, and decision trees are good at processing high-dimensional data, and have excellent performance in sensitivity, specificity, and prediction effect (50). In the future, machine learning technology can be considered to be used in the development and validation of complex models, aiming to construct a frailty risk prediction model for stroke patients with improved predictive performance and greater stability (51, 52).

Predictors could significantly affect the performance of prediction models. As frailty is the consequence of multiple influences, most included studies chose multi-dimensional factors as candidate predictors. The results of this systematic review showed that there were many predictors in the risk prediction models included in the studies. Among them, age, activities of daily living and NIHSS score were the most frequently occurring common predictors. Aging is accompanied by degeneration of multiple systems such as nerves, muscles, and immunity, which is characterized by chronic low-grade inflammation and decreased repair capacity. This reduction in multi-system physiological reserve makes it difficult to recover homeostatic imbalance after the acute stress event of stroke in elderly patients, and it is easy to trigger or accelerate the occurrence of frailty (53). Limitation in activities of daily living reflects a combination of neurological deficits, muscle weakness, balance disturbances, and possibly cognitive decline. Mobility limitation directly leads to disuse syndrome. At the same time, the psychological stress caused by functional dependence further damages muscle and immune function by increasing cortisol (54). The NIHSS score reflects the severity of neurological deficit. The total score is 42 points, and the higher the score is, the more serious the neurological deficit is. This may be because frailty is related to the impairment of brain cell autonomic regulation, and may be accompanied by other functional impairments, such as hemiplegia, speech disorder, and dysphagia, which increase the risk of frailty (55). Therefore, in clinical practice, for stroke patients with advanced age, severe neurological deficit, and early onset of severe ADL dependence, frailty screening and multidisciplinary comprehensive intervention should be carried out as soon as possible.

## 6 Limitations

This systematic review has several limitations: (1) All included studies were conducted in China (2023–2025). Frailty prevalence, stroke management pathways, rehabilitation services, and sociodemographic factors vary substantially across regions and healthcare systems. Therefore, these models cannot be assumed to perform adequately in non-Chinese populations without appropriate external validation. This severely limits the transportability of findings to other countries or ethnic groups. (2) Extreme heterogeneity (*I*^2^ = 90.2%) persisted despite subgroup analysis. This raises questions about the appropriateness of quantitative pooling itself. The pooled estimate should therefore be interpreted as a descriptive summary of reported values rather than a meaningful meta-analytic effect size. Differences in frailty definitions, study designs, model types, validation strategies, predictor sets, and outcome definitions likely contributed. Additionally, correlated AUC estimates from studies developing multiple models were treated as independent. This may lead to overrepresentation of specific datasets and underestimation of uncertainty (i.e., overly narrow confidence intervals). Consequently, the pooled estimate should be interpreted with additional caution, as this statistical dependency is not accounted for., narrative synthesis may be more appropriate for future reviews as evidence accumulates. (3) Four cross-sectional studies were included, which violate the temporal ordering required for true risk prediction. Their AUCs reflect concurrent discrimination rather than prospective validity. As these are diagnostic classification models rather than prognostic prediction models, their inclusion alongside prospective studies further limits interpretability of pooled estimates. While sensitivity analysis suggests limited impact on the pooled estimate, this conceptual distinction should be considered. (4) All included studies were at high risk of bias according to PROBAST, meaning the observed AUC values are likely overestimated. The pooled AUC of 0.87 should be interpreted with caution and does not imply that current models are ready for clinical application. (5) Heterogeneous frailty definitions across studies—ranging from purely physical to multidimensional constructs—limit the conceptual coherence of pooled estimates. While subgroup analysis by instrument partially addresses this, the underlying difference in what is being predicted (physical vs. biopsychosocial frailty) means that model performance is not directly comparable across instruments. This should be considered when applying findings to clinical practice or future research.

## 7 Conclusion

This systematic review summarized 19 studies and 24 frailty risk prediction models for stroke patients, All included studies were from China, and the pooled AUC (0.87) is likely inflated due to high risk of bias. These findings cannot be generalized to non-Chinese populations without external validation and should be interpreted as exploratory. Cross-sectional models in this review assess current frailty status, not future risk. Current evidence does not support clinical use of these models. Therefore, future research must adhere to PROBAST and TRIPOD guidelines, use prospective multi-center designs, handle missing data appropriately, and perform external validation in diverse international settings so as to develop reliable prediction models.

## Data Availability

The original contributions presented in the study are included in the article/supplementary material, further inquiries can be directed to the corresponding author.

## References

[1] Miranda LA Luvizutto GJ Stephan BCM Souza JT Silva TR Winckler FC . Evaluating the performance of the PRISMA-7 frailty criteria for predicting disability and death after acute ischemic stroke. J Stroke Cerebrovasc Dis. (2022) 31:106837. doi: 10.1016/j.jstrokecerebrovasdis.2022.106837 36283237

[2] Ramos-Lima MJM Brasileiro IC de Lima TL Braga-Neto P. Quality of life after stroke: impact of clinical and sociodemographic factors. Clinics (Sao Paulo). (2018) 73:e418. doi: 10.6061/clinics/2017/e418 30304300 PMC6152181

[3] Sun YA Kalpakavadi S Prior S Thrift AG Waddingham S Phan H . Socioeconomic status and health-related quality of life after stroke: a systematic review and meta-analysis. Health Qual Life Outcomes. (2023) 21:115. doi: 10.1186/s12955-023-02194-y 37875951 PMC10599023

[4] Huang YN Yan FH Wang XY Chen XL Chong HY Su WL . Prevalence and risk factors of frailty in stroke patients: a meta-analysis and systematic review. J Nutr Health Aging. (2023) 27:96–102. doi: 10.1007/s12603-023-1879-z, 36806864 PMC12880042

[5] Mennema Å Vliet Vlieland TPM Achterberg WP Oosterveer DM the SCORE-study group. Functioning and recovery during stroke rehabilitation: a comparison between pre-stroke frail and non-frail patients. Eur Geriatr Med. (2023) 14:1343–51. doi: 10.1007/s41999-023-00885-9, 37935943 PMC10754730

[6] Álvarez-Bustos A Carnicero-Carreño JA Sanchez-Sanchez JL Garcia-Garcia FJ Alonso-Bouzón C Rodríguez-Mañas L. Associations between frailty trajectories and frailty status and adverse outcomes in community-dwelling older adults. J Cachexia Sarcopenia Muscle. (2022) 13:230–9. doi: 10.1002/jcsm.12888, 34951157 PMC8818602

[7] Pasquet R Xu M Sylvestre MP Keezer MR. Comparison of three frailty measures for predicting hospitalization and mortality in the Canadian longitudinal study on aging. Aging Clin Exp Res. (2024) 36:48. doi: 10.1007/s40520-024-02706-w, 38418612 PMC10902012

[8] Chen SF Li HH Guo ZN Ling KY Yu XL Liu F . Association between pre-stroke frailty status and stroke risk and impact on outcomes: a systematic review and meta-analysis of 1,660,328 participants. Aging Clin Exp Res. (2024) 36:189. doi: 10.1007/s40520-024-02845-0, 39259235 PMC11390839

[9] Hanlon P Nicholl BI Jani BD Lee D McQueenie R Mair FS. Frailty and pre-frailty in middle-aged and older adults and its association with multimorbidity and mortality: a prospective analysis of 493 737 UK biobank participants. Lancet Public Health. (2018) 3:e323–32. doi: 10.1016/S2468-2667(18)30091-4, 29908859 PMC6028743

[10] Kanai M Noguchi M Kubo H Nozoe M Kitano T Izawa KP . Pre-stroke frailty and stroke severity in elderly patients with acute stroke. J Stroke Cerebrovasc Dis. (2020) 29:105346. doi: 10.1016/j.jstrokecerebrovasdis.2020.105346 33032021

[11] Evans NR Wall J To B Wallis SJ Romero-Ortuno R Warburton EA. Clinical frailty independently predicts early mortality after ischaemic stroke. Age Ageing. (2020) 49:588–91. doi: 10.1093/ageing/afaa004, 31951248

[12] Wæhler IS Saltvedt I Lydersen S Fure B Askim T Einstad MS . Association between in-hospital frailty and health-related quality of life after stroke: the nor-COAST study. BMC Neurol. (2021) 21:100. doi: 10.1186/s12883-021-02128-5, 33663430 PMC7931593

[13] Burton JK Stewart J Blair M Oxley S Wass A Taylor-Rowan M . Prevalence and implications of frailty in acute stroke: systematic review and meta-analysis. Age Ageing. (2022) 51. doi: 10.1093/ageing/afac064, 35352795 PMC9037368

[14] Pinho J Küppers C Nikoubashman O Wiesmann M Schulz JB Reich A . Frailty is an outcome predictor in patients with acute ischemic stroke receiving endovascular treatment. Age Ageing. (2021) 50:1785–91. doi: 10.1093/ageing/afab092 34087930

[15] Kong LN Yang L Lyu Q Liu DX Yang J. Risk prediction models for frailty in older adults: a systematic review and critical appraisal. Int J Nurs Stud. (2025) 167:105068. doi: 10.1016/j.ijnurstu.2025.105068 40184783

[16] Hoogendijk EO Afilalo J Ensrud KE Kowal P onder G Fried LP. Frailty: implications for clinical practice and public health. Lancet. (2019) 394:1365–75. doi: 10.1016/S0140-6736(19)31786-6 31609228

[17] Moons KG de Groot JA Bouwmeester W Vergouwe Y Mallett S Altman DG . Critical appraisal and data extraction for systematic reviews of prediction modelling studies: the CHARMS checklist. PLoS Med. (2014) 11:e1001744. doi: 10.1371/journal.pmed.1001744, 25314315 PMC4196729

[18] Wolff RF Moons KGM Riley RD Whiting PF Westwood M Collins GS . PROBAST: a tool to assess the risk of Bias and applicability of prediction model studies. Ann Intern Med. (2019) 170:51–8. doi: 10.7326/M18-1376 30596875

[19] Page MJ McKenzie JE Bossuyt PM Boutron I Hoffmann TC Mulrow CD . The PRISMA 2020 statement: an updated guideline for reporting systematic reviews. BMJ. (2021) 372:n71. doi: 10.1136/bmj.n71 33782057 PMC8005924

[20] Chen Z Kong X Wang G Zhou L Wang S. Establishment and validation of a prediction model for geriatric frailty syndrome in elderly patients with AIS after treatment. Chinese Journal of Geriatric Heart Brain and Vessel Diseases. (2023) 25:1336–9. doi: 10.3969/ji.ssn.1009-0126.2023.12.025

[21] Ding L Le Y Cheng J Wan X Yu X. Construction and validation of a nomogram prediction model for frailty after stroke. J Clin Nurs. (2023) 22:26–9. doi: 10.3969/j.issn.1671-8933.2023.06.008

[22] Gao X Sun L Wang Y Wang X Wu X Yang L. Construction and verification of a prediction model for frailty in elderly patients with ischemic stroke. Journal of nurses training. (2025) 40:731–8. doi: 10.16821/j.cnki.hsjx.2025.07.010

[23] Jiang X Li M Wang L. Analysis of the influencing factors of frailty in elderly patients with ischemic stroke and construction of risk prediction model. Journal of Bengbu Medical University. (2024) 49:784–9. doi: 10.13898/j.cnki.issn.1000-2200.2024.06.019

[24] Qiao L Wang M Zhao Y Liu Y Gu Y Shi X. Analysis of the factors influencing frailty in elderly ischemic stroke patients based on a Bayesian network. Modern Medical Journal. (2025) 53:515–22. doi: 10.3969/j.issn.1671-7562.2025.04.001

[25] Wang S Jiang H Xu J Wu Y. Construction and validation of frailty risk prediction model in patients with acute stroke. Chongqing Medicine. (2024) 53:3100–7. doi: 10.3969/j.issn.1671-8348.2024.20.011

[26] Zhang L Wang Y Chen C Du Q Pang X. A prospective study of prediction model for frailty after ischemic stroke in elderly patients. Chinese General Practice Nursing. (2024) 22:1398–404. doi: 10.12104/j.issn.1674-4748.2024.08.003

[27] Gao J. Construction of predictive model of frailty inelderly patients with acute cerebral infarction. Henan University. (2024). p. 60.

[28] Chen X. Construction of a frailty prediction model for elderly stroke hospitalized patients based on decision. Yangtze University. (2024). p. 90.

[29] Shen D Li J Teng S Li M Tang X. Development and validation of a nomogram for predicting frailty risk among older patients with Ischaemic stroke. J Clin Nurs. (2024) 34:4186–201. doi: 10.1111/jocn.17627 39710612

[30] Wang J Yuan Y Zhang Y Wang M Yan Y. Assessment of frailty status in patients with acute cerebral infarction and their relationship with serum markers. Am J Transl Res. (2024) 16:6018–28. doi: 10.62347/CWFR7413, 39544753 PMC11558393

[31] Zhong X Liu X. Construction and validation of a risk-prediction nomogram model for frailty in elderly patients with dysphagia after acute cerebral infarction. Chinese Journal of Multiple Organ Diseases in the Elderly. (2023) 22:924–9. doi: 10.11915/j.issn.1671-5403.2023.12.195

[32] Liang Y Dai L Chen J Jiao Y Su M Wu P. Analysis of risk factors for frailty in hospitalized elderly patients with cerebral infarction and construction of a joint prediction model. Journal of Mudanjiang Medical College. (2024) 45:80. doi: 10.13799/j.cnki.mdjyxyxb.2024.04.018

[33] Zhang X Zhang L Sui R. Prediction model for frailty in elderly stroke patients based on logistic regression and artificial neural network. Military. Nursing. (2023) 40:19. doi: 10.3969/j.issn2097-1826.2023.02.003

[34] Zhang X Qin L Chen W Sun Y. A random forest-based multimodal neural electrophysiological index model for predicting frailty risk in elderly patients with stroke. International Medicine and Health Guidance News. (2025) 31:973–8. doi: 10.3760/cma.j.cn441417-20240910-06018

[35] Zhang N. Construction and Validation of Debilitating Risk Prediction Model for Patients With Acute lschemic Stroke. Zhengzhou University. (2024). p. 67.

[36] Li Y. Study of the prediction model for frailty in elderly stroke patients based on decision trees. Lanzhou University. (2024). p. 79.

[37] Qiao L Zhao Y Liu Y Zhao X Shi X Wang M. Effect of prognostic nutritional index and vertebral artery resistance index on frailty prediction model in elderly patients with mild ischemic stroke based on XGBoost-SHAP. Chinese Journal of Prevention and Control of Chronic Diseases. (2025) 33:357–62. doi: 10.16386/j.cjpccd.issn.1004-6194.20240725.0554

[38] Ma Y Wang X Dou L. Frail status of elderly patients with mild ischemic stroke and construction of a prediction risk model based on random forest algorithm. Chinese Journal of Geriatric Heart Brain and Vessel Diseases. (2025) 27:187–91. doi: 10.3969/j.issn.1009-0126.2025.02.014

[39] Hou YG Feng SM Wang SM Zhao YJ Yan L. The construction and validation of a frailty risk prediction model for older adults with lung cancer: a cross-sectional study. Eur J Oncol Nurs. (2023) 64:102316. doi: 10.1016/j.ejon.2023.102316, 37141666

[40] Wang J Kwan RYC Suen LKP Lam SC Liu N. Development and validation of a risk identification model for frailty in stroke survivors: new evidence from CHARLS. BMC Public Health. (2025) 25:2939. doi: 10.1186/s12889-025-24198-7, 40866896 PMC12382056

[41] Kong L Zhang H Jiang P. Construction and validation of nomogram to predict the frailty risk of senile stroke patients. Geriatr Nurs. (2025) 65:103477. doi: 10.1016/j.gerinurse.2025.103477, 40645135

[42] Moons KGM Wolff RF Riley RD Whiting PF Westwood M Collins GS . PROBAST: a tool to assess risk of Bias and applicability of prediction model studies: explanation and elaboration. Ann Intern Med. (2019) 170:W1–w33. doi: 10.7326/M18-1377 30596876

[43] Talari K Goyal M. Retrospective studies—utility and caveats. J R Coll Physicians Edinb. (2020) 50:398–402. doi: 10.4997/jrcpe.2020.409 33469615

[44] Ogundimu EO Altman DG Collins GS. Adequate sample size for developing prediction models is not simply related to events per variable. J Clin Epidemiol. (2016) 76:175–82. doi: 10.1016/j.jclinepi.2016.02.031, 26964707 PMC5045274

[45] Allotey PA Harel O. Multiple imputation for incomplete data in environmental epidemiology research. Curr Environ Health Rep. (2019) 6:62–71. doi: 10.1007/s40572-019-00230-y, 31090043

[46] Aljrees T. Improving prediction of cervical cancer using KNN imputer and multi-model ensemble learning. PLoS One. (2024) 19:e0295632. doi: 10.1371/journal.pone.0295632, 38170713 PMC10763959

[47] Reps JM Ryan P Rijnbeek PR. Investigating the impact of development and internal validation design when training prognostic models using a retrospective cohort in big US observational healthcare data. BMJ Open. (2021) 11:e050146. doi: 10.1136/bmjopen-2021-050146, 34952871 PMC8710861

[48] Efthimiou O Seo M Chalkou K Debray T Egger M Salanti G. Developing clinical prediction models: a step-by-step guide. BMJ. (2024) 386:e078276. doi: 10.1136/bmj-2023-078276, 39227063 PMC11369751

[49] de Jong Y Ramspek CL van der Endt VHW Rookmaaker MB Blankestijn PJ Vernooij RWM . A systematic review and external validation of stroke prediction models demonstrates poor performance in dialysis patients. J Clin Epidemiol. (2020) 123:69–79. doi: 10.1016/j.jclinepi.2020.03.015, 32240769

[50] Zhou Y Liu Y Zhang Y Zhang Y Wu W Fan J. Identifying non-linear association between maternal free thyroxine and risk of preterm delivery by a machine learning model. Front Endocrinol (Lausanne). (2022) 13:817595. doi: 10.3389/fendo.2022.817595, 35282469 PMC8907667

[51] Severson KA Chahine LM Smolensky LA Dhuliawala M Frasier M Ng K . Discovery of Parkinson's disease states and disease progression modelling: a longitudinal data study using machine learning. Lancet Digit Health. (2021) 3:e555–64. doi: 10.1016/S2589-7500(21)00101-1 34334334

[52] Schwartzmann B Dhami P Uher R Lam RW Frey BN Milev R . Developing an electroencephalography-based model for predicting response to antidepressant medication. JAMA Netw Open. (2023) 6:e2336094. doi: 10.1001/jamanetworkopen.2023.36094, 37768659 PMC10539986

[53] Pilotto A Brass C Fassbender K Merzou F Morotti A Kämpfer N . Premorbid frailty predicts short and long-term outcomes of reperfusion treatment in acute stroke. J Neurol. (2022) 269:3338–42. doi: 10.1007/s00415-022-10966-7, 35039903

[54] Cui Y Xiang L Zhao P Chen J Cheng L Liao L . Machine learning decision support model for discharge planning in stroke patients. J Clin Nurs. (2024) 33:3145–60. doi: 10.1111/jocn.16999, 38358023

[55] de Berker H de Berker A Aung H Duarte P Mohammed S Shetty H . Pre-stroke disability and stroke severity as predictors of discharge destination from an acute stroke ward. Clin Med (Lond). (2021) 21:e186–91. doi: 10.7861/clinmed.2020-0834, 33762385 PMC8002797

